# Microwave Analysis of Scattered and Absorbed Powers of Semiconductor and Metamaterial Cylinder Structures

**DOI:** 10.3390/ma12020265

**Published:** 2019-01-15

**Authors:** Juozas Bučinskas, Raimondas Pomarnacki, Darius Plonis, Šarūnas Paulikas, Giedrius Tušinskis, Liudmila Nickelson

**Affiliations:** 1 Chemical Physics Institute, Vilnius University, Sauletekio str. 9, 10222 Vilnius, Lithuania; juozas.bucinskas@ff.vu.lt (J.B.); giedriustusinskis@gmail.com (G.T.); 2 Department of Electronic Systems, Vilnius Gediminas Technical University, Naugarduko str. 41, 03227 Vilnius, Lithuania; raimondas.pomarnacki@vgtu.lt (R.P.); liudmila.nickelson@vgtu.lt (L.N.); 3 Department of Computer Science and Communications Technologies, Vilnius Gediminas Technical University, Naugarduko str. 41, 03227 Vilnius, Lithuania; sarunas.paulikas@vgtu.lt

**Keywords:** electromagnetic (EM) analysis, microwave, metamaterial cylinder, semiconductor cylinder, semiconductor-metamaterial arrays, parallel and perpendicular polarizations, EM scattering, EM reflection, absorbed power, scattered power

## Abstract

Here is presented our numerical investigations based on the rigorous solution of the Maxwell’s equations for analyses of absorbed and scattered powers of a semiconductor-metamaterial array with a window defect. The array structure consists of a finite set of infinite parallel, circular cylinders that can be made of the different lossy and/or lossless isotropic materials. We used our developed computer code, which allowed us to consider an array consisting of an arbitrary number of cylinders. According to our code, cylinders can be located at different distances and have differing diameters. There is a limitation: Cylinders should not cross each other. We numerically examined two cylindrical arrays with electromagnetic (EM) band-gap (EBG) defects. The absorbed and scattered powers were analyzed there for parallel and perpendicular polarizations of the incident microwave. We investigated dependencies on the operating frequency and the radius (R) of an arc of the arranged thirteen n-Si cylinders with the low semiconductor specific resistivity of 0.5, 2, and 10 Ω∙m. We have discovered that the arrays may have features of a waveguide or a microwave reflector.

## 1. Introduction

Scattering, diffraction, and propagation problems concerning cylindrical obstacles play a significant role, even today. There are functional devices of great importance, such as antennas, switches, electromagnetic (EM) band gap structures with multiple applications, reflectors, polarizers, resonators, splitters, and so on made on the basis of cylindrical arrays [1,2,3,4,5]. 

Many cylinders arranged in some order usually name a periodic (with the same space in between) or aperiodic array of cylinders. The cylinders could be made of semiconductors, insulators, conductors, metamaterials, and other materials that can be characterized by the real or complex permittivity and permeability. An array of cylinders can have the forbidden frequency band and it can be also named a cylindrical EM band gap (EBG) structure.

A set of cylinders can be called an ideal array of cylinders, which are the ones that are placed in the regular order and made of material with the same constitutive parameters. A set can be named the array with defects, when one or more cylinders in the array are removed or some cylinders’ locations, dimensions, shapes, and materials of which they are made are changed.

In [1] the results of measurements and simulations of a cylindrical EBG antenna are presented. The simulations were fulfilled by a computer code using the finite-difference time-domain method. The analyzed structure was composed by infinite long ceramic rods arranged in the concentric circle.

It is popular enough to have an array of similar obstacles in the microwave range and thus to name also a photonic (or electromagnetic) crystal by analogy with structures in the optical range [2]. In the last article, it is mentioned that splitters, couplers, and filters can be performed on the basis of photonic crystal waveguide devices (PCWD). The analyzed PCWD is formed by the infinitely-long identical, circular cylinders. The wave propagation is controlled by removing some cylinders (i.e., there is a periodic cylinder array with defects). The reflected and transmission powers vice versa to the wavelength in free space are presented in [2]. 

Analysis of EBG structures of M infinite parallel circular cylinders, arranged on each of the N concentric or eccentric circles at the oblique incident plane wave, is given in [3]. The rigorous semi-analytical method based on the cylindrical Floquet mode expansion is given in the mentioned article. The resonance and stopband regions of the transmission spectra and the EM field distributions are presented in [3].

In [4,5], radiation patterns, transmission coefficients, and the directivity of cylindrical EBG structures, with and without defects, are given. The EBG structures consisted of several layers of metallic or dielectric cylinders. The articles mention that on the basis of the EBG structure, beam-switching and directive and multi-band reconfigurable antennas can be created.

When the above-mentioned devices contain metamaterial elements then they can acquire novel technical possibilities [6,7]. The highly unusual value of metamaterial constitutive parameters may invoke previously unknown physical phenomena, such as EM cloaking, space coordinate transformation for invisibility cloaks, and radar illusion. New EM effects, when creating devices, are easier to discover by theoretical modeling in comparison to the experimental and technological research. The boundary problem solutions and calculations concerning the EM wave scattering caused by cylinders are given in [8,9,10,11,12,13]. The scattering by a single cylinder is presented in [8,9]. In [8], the hybrid method of moments and the Monte Carlo simulation to investigate the single cylinder made of semiconductor (Ge) or metal (Al) is demonstrated. In [9], calculations by way of the rigorous electrodynamical method, through the TE- and TM-wave potentials, for the analyses of microwave scattering problems are presented. In [10], the modification of the method of momentums and three versions of interpolation schemes for solving the EM scattering problem of dielectric cylinders are given. In [11], a scattering problem formulation for conductor cylinders with the dielectric or metamaterial coating is presented. The formulation was based on the expansion of the EM field by Bessel functions in local coordinates. The incident EM wave is the plane TM polarized one. In the last article, the scattering and forward-scattering cross sections of several cylinders, with some fixed values of permittivity and permeability, is numerically investigated. In [12], an accurate method, based on the recursive transition-matrix algorithm, for analyzing the plane wave scattering from a 2D periodic circular cylindrical array with defects is demonstrated. The numerical results are given for the array of lossless dielectric cylinders. In [13], properties of 2D photonic crystals that were formed by parallel infinite homogeneous dielectric cylinders are considered. 

The solution of the Maxwell’s equation for the determination of the Poynting vector for two array structures, which are composed of the infinite parallel cylinders, is given in our previous article [14]. This solution allows one to determinate the directional energy flux density (the rate of energy transfer per unit area) of an EM field in any point. In [14], angular dependency of the radial component P_ρ_, of the Poynting vector, was investigated. The component P_ρ_ dependency on an arc radius of the array structure and the frequency of the incident wave at the fixed distance from the arc center were investigated. The distance was taken 100 mm from the arc center along the incident wave propagation (φ = 0) and in the opposite direction (reflected field, φ = π).

For the development of microwave devices, it is important to know the integrated features such as the total scattered and absorbed powers from the entire array structure. We have developed algorithms to determine these powers. The present article is a continuation of the work in [14]. We proposed here an algorithm on the base of the rigorous solution of Maxwell’s equations for the analysis of total scattered and absorbed energy per one period of the oscillations for unit length of a cylindrical array. In our article, two approaches and main formulae for the determination of the scattered power are given. The proposed algorithms allow for the reduction of the order of a system of linear equations, which is obtained after satisfying the boundary conditions, twice in comparison with the analogical equation system in [14]. For this reason, the computation time is greatly reduced. The method we have proposed allows for the investigation of arrays containing perfect electric conductor (PEC), isotropic dielectric, uniaxial dielectric, or ferrite cylinders in the same arrays using the same algorithm.

In the present article, considered arrays of thirteen semiconductor cylinders and one metamaterial cylinder may be treated as EBG arrays with defects. The defects are defined as the central metamaterial cylinder, which has the different radius and constitutive parameters as well as a π-window opening. The last defect shows that the array structures have empty space between successions of periodically alternating cylinders. The array characteristics are different dependent on the location of the window opening.

We verified our algorithm by comparing our calculations with some special cases when the task parameters, such as as the cylinder radius, the distance between cylinders, the angle of incidence of microwave, and the permittivity, have the limit values.

In our boundary problem for finding the solution of the linear equation system, the FORTRAN code standard routines of the International Mathematics and Statistics Library (IMSL) was applied. We utilized full-featured data analysis software, OriginPro 9.0.0, to create graphs. 

## 2. Materials and Methods

**Figure 1:**
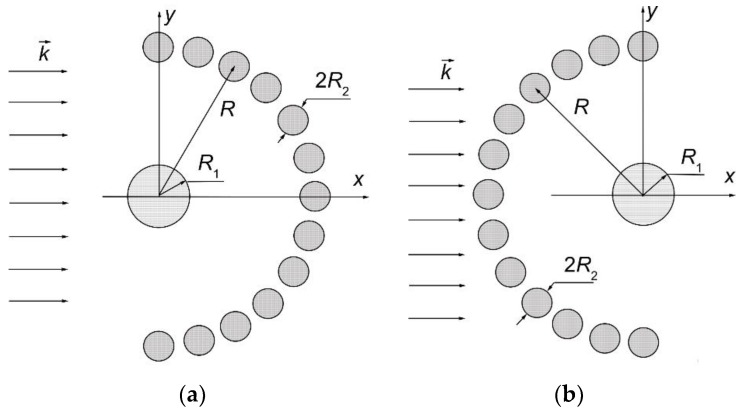
Lossy metamaterial-semiconductor array structures when: (**a**) Thirteen semiconductor cylinders are placed on the right side of the central metamaterial cylinder; (**b**) the cylinders are placed on the left side of the central metamaterial cylinder.

We present here the analysis of microwave absorbed and scattered powers of the multi-cylindrical array. The array consisted of a finite number of infinite cylinders. There are N parallel cylinders that are placed in an isotropic homogeneous medium (e.g., air with the relative permittivity ε = 1 and the relative permeability µ = 1) (Figure 1). The identical silicon cylinders were placed on the arc. The cylinder was located in the center of arc, and was made from metamaterial UCSD30815 [17]. There are graphs of the dependence of the permittivity and permeability metamaterial on the operating frequency in [17]. These graphs were translated by a computer program into a set of numerical data, which, at the appropriate frequencies, were used in our calculations.

In the investigated structures, the permittivity of semiconductor and metamaterial cylinders were much higher than air, which filled the spaces between the cylinders (real part of the relative permittivity of air is 1 and our semiconductor was about 12); for this reason there was no problem with the dielectric contrast. Structures presented in Figure 1 can be accurately calculated by an almost analytical method.

The radii of cylinders are R_s_, s = 1, …, N, and the relative material parameters of every cylinder are ε_s_, μ_s_. We denoted with index “s” the cylinder for which the boundary conditions are written on its interface, and the other cylinders, which only participated in the boundary conditions, with index “p”. The axis of every cylinder was parallel to the z axis of the Cartesian coordinate system. The designation of every cylinder center was ${\vec{r}}_{s}$, s = 1, …, N at the plane z = 0. There was a valid requirement for:

$$|{\vec{r}}_{p}-{\vec{r}}_{s}|\geq R_{p}+R_{s},p\neq s$$

This means that all cylinders do not cross each other and no single cylinder can be inside of another one. Cylinders can only touch each other by their external surfaces. Practical realization of considered structures was not complicated because the height of cylinders can be taken with finite dimensions. The height of cylinders, in practice, should be about an order greater than the wavelength of the incident EM wave. It is clear, from well-known EM field theory knowledge, that if the height of a cylinder is more than ten times larger than the wavelength of the incident wave then, in a solution, the approximation that the cylinders are infinite can be taken and the boundary conditions at the ends of the cylinders can be ignored. This approach simplifies the task being considered and gives accurate calculation results.

The external EM field consists of an incident EM wave and scattered EM waves of all cylinders of the array. The EM field inside and outside of the cylinders are convenient to describe by way of the transverse electric (TE) mode and the transverse magnetic (TM) mode potentials [14,15]. The solution of the Maxwell’s equations in the cylinder area after the Fourier transformation, with respect to the coordinate z, can be written in the form as is given in [14].

The absorbed energy per unit length and per period of single cylinder,$W_{j}^{a}$, j = 1, …, N, can be computed in the same way as it was described in [15]. The full absorbed energy one obtains after summing is in respect to cylinder number j. The scattering energy can be calculated in two ways. The first way allows us to easily determine the scattering energy, but does not allow for the estimation of the contribution of each cylinder (Equations (1) and (2)). The second way is more complicated (Equation (4)), but it allows for the determination of the contribution from each cylinder.

In the first way, we surround a cylinder array by a fictitious cylindrical surface of radius R_c_, with the center in the point ${\vec{r}}_{c}$ and the parallel axis to the other cylinders. The electric field on the inner side of the fictitious cylinder consists of reradiated fields of cylinders. The outer side of the fictitious cylinder the EM field may be treated as radiated by the fictitious cylinder, with unknown amplitudes of cylindrical waves. Equality of tangent components of the electric field, on the fictitious cylinder surface, yields formulae for amplitudes of TM and TE waves radiated by the fictitious cylinder:

(1) $${\bar{A}}_{m}=\sum_{p=1}^{N}\sum_{n=-M_{p}}^{M_{p}}A_{p,n}^{sc}J_{m-n}(\beta r_{pc})exp(-i(m-n)\varphi_{pc})$$

(2) $${\bar{B}}_{m}=\sum_{p=1}^{N}\sum_{n=-M_{p}}^{M_{p}}B_{p,n}^{sc}J_{m-n}(\beta r_{pc})exp(-i(m-n)\varphi_{pc})$$

where β = (k^2^εµ − h^2^)^1/2^, h = k_z_·(εµ)^1/2^ is the value of the Fourier parameter, ε is the relative permittivity of medium, µ is the relative permeability of medium, and k_z_ is the longitudinal component z of wave vector k of the incident EM wave. When the incident wave is a monochromatic plane wave, then the formulae are valid at the angle θ between the wave vector k and the z axis is 0 < θ < π and k_z_ = kcos(θ). The wave vector also can be expressed as k = ω·(ε_0_µ_0_)^1/2^, where ω is an angular frequency of the incident wave, ε_0_ is the vacuum permittivity, and µ_0_ is the vacuum permeability. 

The scattering energy can be found after the defining of amplitudes ${\bar{A}}_{m}$ and ${\bar{B}}_{m}$ by using method [15]. It should be mentioned that the range of variation of index m has to exceed a range of variation of index n at least twice in order to obtain a reasonable result. Formulaes (1) and (2) allow us to calculate the common scattered energy by the simplest way.

The second way is to find average scattered energy over the oscillation period for the unit length of a cylinder with the radius ρ for what it is necessary to calculate the integral:

(3) $$W_{j}^{s}=\frac{1}{2}\int_{0}^{1}dz\int_{0}^{2\pi }\left([\vec{E},{\vec{H}}^{*}]{\vec{n}}_{\rho }\right)\rho d\varphi$$

where ${\vec{n}}_{\rho }$ is the unit vector in the radial direction of the cylindrical coordinate system, superscript * means the complex conjugate’s operation, $\vec{E}$ and $\vec{H}$ is the scattered fields on the outer side of surface of j-th cylinder, where index j can take values from 1 until the quantity of cylinders N. The index p corresponds to the number of cylinders in the array and it can also have values from 1 to N at the mandatory condition j ≠ p. Thus, the total scattered energy of the unit length array structure, per period of the oscillations, is equal to sum of the integrals in Equation (3). The summation is performed for each of the cylinders. After applying Graf’s additional theorem [16], and the integration of the contribution of the cylinder with the number j, we get relation:

(4) $$W_{j}^{s}=-\frac{1}{2}\frac{ik\varepsilon^{*}z_{j}}{Z_{0}}\beta^{*}\beta \{\sum_{m=-M_{j}}^{M_{j}}[A_{j,m}^{sc}A_{j,m}^{sc*}H_{m}(z_{j}){H^{\prime }}_{m}^{*}(z_{j})+A_{j,m}^{sc}{J^{\prime }}_{m}^{*}(z_{j})H_{m}(z_{j})\Phi^{*}(m,A_{}^{sc},j)\;+A_{j,m}^{sc*}J_{m}(z_{j}){H^{\prime }}_{m}^{*}(z_{j})\Phi (m,A_{}^{sc},j)]+\sum_{m=-\infty }^{\infty }J_{m}(z_{j}){J^{\prime }}_{m}^{*}(z_{j})\Phi (m,A_{}^{sc},j)\Phi^{*}(m,A^{sc},j)\}\;+\frac{1}{2}iZ_{0}k\mu \;z_{j}^{*}\beta^{*}\beta \{\sum_{m=-M_{j}}^{M_{j}}[B_{j,m}^{sc}B_{j,m}^{sc*}{H^{\prime }}_{m}(z_{j})H_{m}^{*}(z_{j})+B_{j,m}^{sc}J_{m}^{*}(z_{j}){H^{\prime }}_{m}(z_{j})\Phi^{*}(m,B^{sc},j)\;+B_{j,m}^{sc*}{J^{\prime }}_{m}^{}(z_{j})H_{m}^{*}(z_{j})\Phi (m,B_{}^{sc},j)]+\sum_{m=-\infty }^{\infty }{J^{\prime }}_{m}(z_{j})J_{m}^{*}(z_{j})\Phi (m,B_{}^{sc},j)\Phi^{*}(m,B_{}^{sc},j)\}\;+\frac{1}{2}\left(\beta^{2}h^{*}–\beta^{*}{}^{2}h\right)\{\sum_{m=-M_{j}}^{M_{j}}[A_{j,m}^{sc}B_{j,m}^{sc*}mH_{m}(z_{j})H_{m}^{*}(z_{j})+mJ_{m}(z_{j})H_{m}^{*}(z_{j})B_{s,m}^{*}\Phi (m,A_{}^{sc},j)\;+mJ_{m}^{*}(z_{j})H_{m}(z_{j})A_{j,m}^{sc}\Phi^{*}(m,B_{}^{sc},j)]+\sum_{m=-\infty }^{\infty }mJ_{m}(z_{j})J_{m}^{*}(z_{j})\Phi (m,A_{}^{sc},j)\Phi^{*}(m,B_{}^{sc},j)\}$$

here noted $\Phi (m,Q^{sc},j)=\sum_{\begin{array}{l} p=1\\ p\neq j \end{array}}^{N}\sum_{n=-M_{p}}^{M_{p}}Q_{p,n}^{sc}H_{m-n}(z_{pj})e^{-i(m-n)\varphi_{pj}}$ and $z_{pj}=\beta r_{pj}$, $Q^{sc}$ is the name of the set of scattered wave amplitudes; the amplitudes $Q^{sc}$ can have values $A^{sc}$ or $B^{sc}$, $z_{j}=\beta R_{j},$ $Z_{0}=\sqrt{\frac{\mu_{0}}{\varepsilon_{0}}}=120\pi$, and $\beta =k\sqrt{\varepsilon \mu }$; φ_pj_ is the polar angle of the vector ${\vec{r}}_{p}-{\vec{r}}_{j}$, where indexes p and j can take values from 1 to N at j ≠ p. The derivative of function with respect to its argument is denoted by the prime symbol ( ′ ), for example, $J_{m}(z_{j})$ is the Bessel function with an argument $z_{j}=\beta R_{j}$, ${J^{\prime }}_{m}(z_{j})$ is the first derivative of the Bessel function, $J_{m}^{*}(z_{j})$ is the complex conjugate of the Bessel function, ${J^{\prime }}_{m}^{*}(z_{j})$ is the complex conjugate value of the first derivative of the Bessel function, and $H_{m}(z_{j})$ is the Hankel function. The same designations are used for this function, for example, ${H^{\prime }}_{m}(z_{j}),H_{m}^{*}(z_{j}),{H^{\prime }}_{m}^{*}(z_{j})$, respectively, are the first derivative, complex conjugate, and complex conjugate of the derivative of the Hankel function.

We can identify the contribution of every cylinder of the array into the common scattered energy by using relation (4). Sometimes it may be useful, for example, when there is need to evaluate the contribution of a single cylinder of the array.

## 3. Results

Here are investigated the total absorbed W^a^ and scattered W^s^ powers of two arrays that are presented in Figure 1a,b. We give here the total scattered and absorbed energy per one period of the oscillations and per unit length of array structure dependencies on the arc radius R, frequency f, and polarization of the incident microwave at three values of the specific resistivity of the semiconductor material.

**Figure 2:**
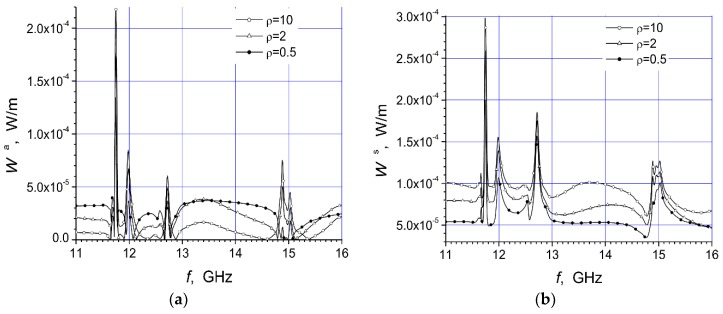
Dependencies of absorbed power (**a**) and scattered power (**b**), of the array Figure 1a, on the frequency of the parallel polarized incident wave at R = 12 mm.

**Figure 3:**
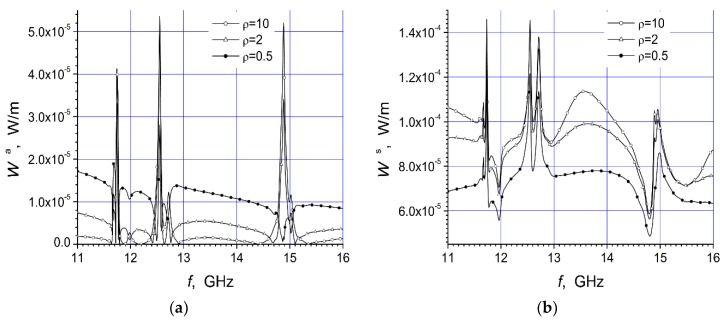
Dependencies of absorbed power (**a**) and scattered power (**b**), of the array Figure 1b, on the frequency of the parallel polarized incident wave at R = 12 mm.

**Figure 4:**
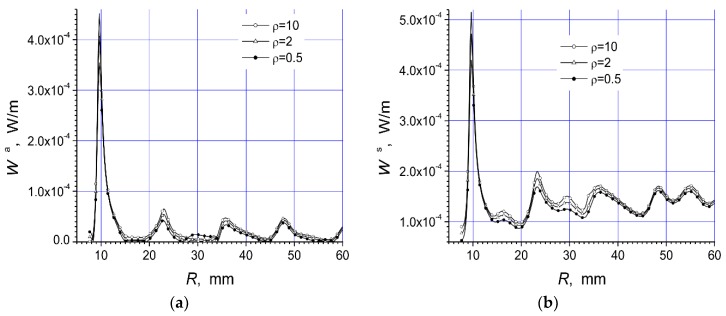
Dependencies of absorbed power (**a**) and scattered power (**b**), of the array Figure 1a, on the arc radius R at the parallel polarized incident wave with f = 12 GHz.

**Figure 5:**
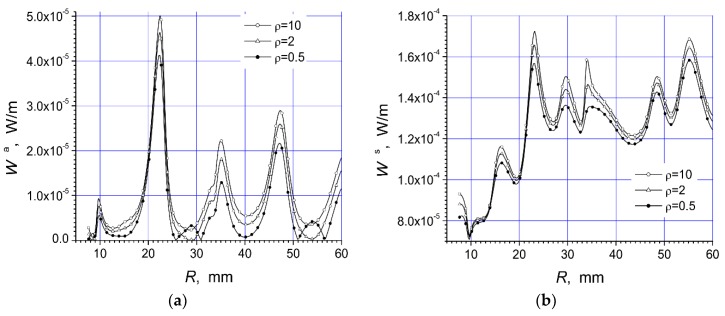
Dependencies of absorbed power (**a**) and scattered power (**b**), of the array Figure 1b, on the arc radius R at the parallel polarized incident wave with f = 12 GHz.

**Figure 6:**
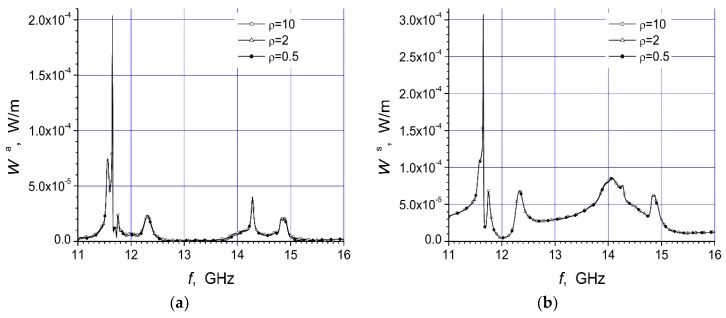
Dependencies of absorbed power (**a**) and scattered power (**b**), of the array Figure 1a, on the frequency of the perpendicular polarized incident wave at R = 12 mm.

**Figure 7:**
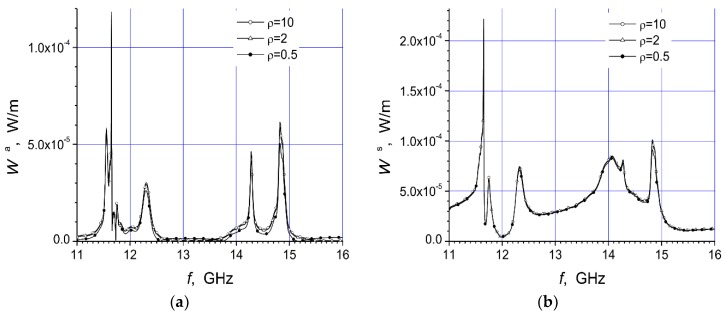
Dependencies of absorbed power (**a**) and scattered power (**b**), of the array Figure 1b, on the frequency of the perpendicular polarized incident wave at R = 12 mm.

The magnitudes of unit length of array absorbed energy (W^a^) and scattered energy (W^s^) per period are presented in watt/meter. In this work we present two arrays consisting of fourteen cylinders. The central cylinder of metamaterial was placed in the origin of the coordinate system and the identical semiconductor n-Si cylinders were equidistantly placed on the arc with radius R (Figure 1). The calculation results for the array Figure 1a are presented in Figure 2a, Figure 3a, Figure 4a, Figure 5a, Figure 6a and Figure 7a. The calculations for the array Figure 1b are shown, respectively, in b figures. The radius R of the arc where the semiconductor cylinders are located was taken, in our calculations, to be equal to R = 12 mm (Figure 2, Figure 3, Figure 6 and Figure 7). The operation frequency was equal to f = 12 GHz in Figure 4 and Figure 5. The radius R_2_ of every n-Si cylinder was 1 mm. The radius R_1_ of the central metamaterial cylinder was 4 mm (Figure 2, Figure 3, Figure 4, Figure 5, Figure 6 and Figure 7). The metamaterial UCSD30815 complex permittivity (ε_1_) and permeability (µ_1_) dependencies on the frequency were taken from Figure 6 and Figure 7 of [17].

The semiconductor n-Si material was the dispersive lossy material. The n-Si permittivity was $\varepsilon_{s}=11.8-i/\left(\omega \varepsilon_{0}\rho \right)$ and the permeability was µ_s_ = 1. Here ρ is the specific resistivity of n-Si material. The specific resistivity of the n-Si was taken as 0.5, 2, or 10 Ω∙m.

The first four Figure 2, Figure 3, Figure 4 and Figure 5 refer to the parallel polarization of the incident microwave. The last three Figure 5, Figure 6 and Figure 7 refer to the perpendicular polarization of the incident EM wave.

In Figure 2 and Figure 3, the total absorbed W^a^ and scattered W^s^ powers of the arrays Figure 1a,b, respectively) are shown, when the incident microwave has the parallel polarization. We see that the absorption resonance peaks for both arrays appeared at different frequencies. The resonance peaks had different amplitudes. Here are presented results which we have got using frequency step 0.01 GHz. These resonances were the dimensional ones. For this reason, the resonances were most dependent on the cylinder radius and distances between cylinders. We used pretty low n-Si specific resistivity values because we were interested as to what impacts carrier concentration changes have on array structure characteristics.

The first resonance of the absorption power of array Figure 1a occurs at the frequency ~11.75 GHz and it has a very large amplitude in comparison with other resonances (Figure 2). Thus, the rotation of the array structure around the metamaterial cylinder, on the π radian, changed the distribution of peak values and shifted the resonance peaks to other frequencies (Figure 2 and Figure 3). The reason for this phenomenon is quite obvious because the incident wave length for frequency f = 11.75 GHz was 2.55 cm. The last value was almost the same as the length of the array section in the direction of the y axis. The maximum distance between the centers of the semiconductor cylinders of the structure along the y axis was 2.4 cm. The maximum distance between the outer points of the structure along the y axis was 2.6 cm because the radius of the n-Si cylinders was 0.1 cm. So, there can be the dimensional resonance of scattering problem on the certain frequencies.

It is important to note that there was strong dependency of the absorbed and scattered powers on the semiconductor specific resistivity at some frequency intervals (e.g., 11–11.6 GHz and 13.2–14.8 GHz). The last dependency can be used for a non-contact method of the semiconductor specific resistivity determination. On the other hand, it was possible to control the specific resistivity of the semiconductor cylinders (e.g., by the light radiation or temperature changes), and change, in this way, the scattered or absorbed powers of the array structure in the pointed frequency intervals.

We can see that the maximum absorption of array structure Figure 1a happened at f ~ 11.75 GHz, and array Figure 1b at f ~ 12.6 GHz and 14.9 GHz. We do not look for extreme values of absorption and scattering powers, because the frequency step was not small enough (in our calculations it was 0.01 GHz), so values in figures should be treated with caution.

There were three significant resonances of absorbed power in Figure 3a. The largest scattered power from array Figure 1b was found at 11.75, 12.5, and 14.9 GHz (i.e., at the same frequencies as the resonances of absorbed power). The comparison of Figure 2b and Figure 3b shows that the case presented in Figure 2b provides the perspective for the non-contact resistivity determination, because the scattered power dependency on the specific resistivity differed markedly throughout the frequency range 13–14.5 GHz.

In Figure 4a and Figure 5a, the absorbed power on the arc radius R for both arrays in Figure 1 are presented.

We see that the absorbed power was significantly dependent on the distance between the metamaterial cylinder and the semiconductor cylinders. The absorption power of array Figure 1a was roughly one order of magnitude lower than of array Figure 1b.

Moreover, it was possible to place the semiconductor cylinders on the distance R, when there was the minimum value W^a^, in order to reduce the absorbed power by the system. The first absorbed power resonance was observed at R ~ 10 mm for the first array (Figure 1a). This resonance was very thin and high in comparison with all other resonances. It can be mentioned that detailed investigations show that the transmission of the microwave through this array structure was minimal, and the reflected wave had the maximal value at R ~ 10 mm. The scattered and absorbed powers of array Figure 1a were the largest at the same frequency because there was the first dimensional resonance.

The values of W^a^ and W^s^ were not significantly altered with the growing of distance R between the metamaterial cylinder and the semiconductor cylinders (Figure 4a,b) when R > 15 mm. We can see in Figure 4a and Figure 5a that the absorption of structures was minimal at the distance R ~ 55 mm. This point was at the edge of the region. It was quite obvious that, for dimensional resonances, re-radiated waves were responsible. There the height of absorbed power resonances diminished with increasing distance between cylinders, but scattered power led into the more complicated way because many re-radiations occur.

The behavior of scattered power (Figure 4b and Figure 5b) was highly notable. The scattered power of the array Figure 1b increased with the increasing of distance R and had many resonance peaks. This was in accordance with the wave nature of the phenomenon.

We noted the same characteristics when the incident microwave had the perpendicular polarization.

In Figure 6 and Figure 7 the dependencies of absorbed and scattered powers for both array structures (Figure 1) are shown. We see that the microwave with the perpendicular polarization weakly reacted on the geometric difference of array structures. In addition, the specific resistivity of the semiconductor did not impact significantly on the absorbed and reflected powers when the incident microwave had the perpendicular polarization. The maximum scattering and absorption of both arrays appeared at ~11.6 GHz, and slightly differed from the parallel polarization case.

The behavior of curves W^a^ and W^s^ for both array structures (Figure 1) was similar. The minimum absorbed and scattered power, in the vicinity of f ~ 12 GHz, had the feature that structure could be applied as a waveguide structure along the axis *x* (see, Figure 1).

The comparison of the dependencies of W^a^ and W^s^ for both polarizations (Figure 2, Figure 3, Figure 6 and Figure 7) showed significant differences in the characteristics. We see that the mentioned dependencies can be increased and decreased in a complex way at different frequency ranges.

## 4. Conclusions

A new algorithm has been developed and tested for the known, from literature, limit values for our investigated array structures. The algorithm was used for analyses of the absorbed and scattered powers for the arrays consisting of lossy or lossless cylinders. Numerical investigations for two arrays are presented here. The arrays contained the central metamaterial cylinder, with radius R_1_ = 4 mm, and thirteen semiconductor cylinders, with radius R_2_ = 1 mm. Semiconductor cylinders were located on the semicircle arc with radius R, which was changed in calculations within interval range 6–60 mm. Calculations were carried out in the frequency range 11–16 GHz for the parallel and perpendicular polarized incident microwaves, at three values of semiconductor n-Si specific resistivity (0.5, 2, and 10 Ω∙m).

We found resonance maximum values (peaks) of absorbed and scattered powers at different frequencies. These peaks had different amplitudes. These resonance values of powers arose due to the dimensional resonance in our investigated structures. These resonances were mostly dependent on the cylinder radius and the distances between cylinders. Numerical analysis of the total absorbed and scattered powers indicates that the arrays may have properties of a waveguide or a reflector. These properties were determined mainly by the distance between the metamaterial and semiconductor cylinders, at the fixed sizes of the cylinders. The maximum absorption and reflection properties of the arrays appeared in approximately the same frequency ranges. The absorption and scattering effects were poorly manifested for a microwave with the perpendicular polarization. The powers were highly dependent on the semiconductor specific resistivity, at some frequency intervals, only if an incident microwave had the parallel polarization. This fact can be used to control the absorbed and scattered powers’ value by way of changing the semiconductor specific resistivity. As it is known, the semiconductor specific resistivity can be changed under the influence of light (and/or heat). Consequently, controlled microwave devices can be created on the basis of the phenomenon.
